# Ratio of Trunk to Leg Volume as a New Body Shape Metric for Diabetes and Mortality

**DOI:** 10.1371/journal.pone.0068716

**Published:** 2013-07-10

**Authors:** Joseph P. Wilson, Alka M. Kanaya, Bo Fan, John A. Shepherd

**Affiliations:** 1 University of California Berkeley-University of California San Francisco Graduate Program in Bioengineering, San Francisco, California, United States of America; 2 Radiology & Biomedical Imaging, University of California San Francisco, San Francisco, California, United States of America; 3 Department of Medicine, University of California San Francisco, San Francisco, California, United States of America; Sapienza, University, Italy

## Abstract

**Background:**

Body shape is a known risk factor for diabetes and mortality, but the methods estimating body shape, BMI and waist circumference are crude. We determined whether a novel body shape measure, trunk to leg volume ratio, was independently associated with diabetes and mortality.

**Methods:**

Data from the National Health and Nutritional Examination Survey 1999–2004, a study representative of the US population, were used to generate dual-energy X-ray absorptiometry-derived trunk to leg volume ratio and determine its associations to diabetes, metabolic covariates, and mortality by BMI category, gender, and race/ethnicity group.

**Results:**

The prevalence of pre-diabetes and diabetes increased with age, BMI, triglycerides, blood pressure, and decreased HDL level. After adjusting for covariates, the corresponding fourth to first quartile trunk to leg volume ratio odds ratios (OR) were 6.8 (95% confidence interval [CI], 4.9–9.6) for diabetes, 3.9 (95% CI, 3.0–5.2) for high triglycerides, 1.8 (95% CI, 1.6–2.1) for high blood pressure, 3.0 (95% CI, 2.4–3.8) for low HDL, 3.6 (95% CI, 2.8–4.7) for metabolic syndrome, and 1.76 (95% CI, 1.20–2.60) for mortality. Additionally, trunk to leg volume ratio was the strongest independent measure associated with diabetes (P<0.001), even after adjusting for BMI and waist circumference. Even among those with normal BMI, those in the highest quartile of trunk to leg volume ratio had a higher likelihood of death (5.5%) than those in the lowest quartile (0.2%). Overall, trunk to leg volume ratio is driven by competing mechanisms of changing adiposity and lean mass.

**Conclusions:**

A high ratio of trunk to leg volume showed a strong association to diabetes and mortality that was independent of total and regional fat distributions. This novel body shape measure provides additional information regarding central adiposity and appendicular wasting to better stratify individuals at risk for diabetes and mortality, even among those with normal BMI.

## Introduction

Body shape is a known risk factor for mortality and diabetes, which is a major global health problem associated with reduced life span, increased morbidity, and significant financial burdens on individuals and health care systems [1], [2]. Body mass index (BMI), an indicator of overall adiposity, and waist circumference, an indicator of central adiposity, are crude measures used to characterize body fatness and are associated with diabetes risk [3], [4], [5], [6]. Typically, those with high BMI or waist circumference are considered to have elevated risk for diabetes and metabolic covariates [3], [7], [8]. However, Carnethon et al recently found that mortality rates were higher in adults with normal BMI at the time of incident diabetes than those individuals who were overweight or obese (by BMI) [9].

Total body volume is the solid volume of an individual and the metric used to measure body density and estimate body composition [10]. Modern work by Behnke et al. draws upon Archimedes’ Principle to characterize obesity using underwater weighing [11]. More than half of a century ago, Siri and Brozek created equations that related body density to total percent fat [12], [13]. These same equations are still used today in water and air displacement devices to provide an estimate of whole body percent fat. Unfortunately, these displacement methods make many assumptions about internal voids that contain air (as in the lungs) or other trapped gasses (as in the stomach or small intestines) and cannot measure volume on a regional level (as in the trunk, arms, or legs) [14].

Dual energy X-ray absorptiometry (DXA) is a highly prevalent medical imaging modality. Typical outputs of a DXA scan report include whole body and regional measures of fat mass, lean mass, bone mineral content (BMC), and bone mineral density. In the last ten years, ratios of regional DXA mass compartments (like trunk to peripheral fat mass and android to gynoid fat mass) have also been used to stratify risk for metabolic diseases [15], [16]. With more than 30,000 systems in the United States and 50,000 systems worldwide, a high test-retest reliability (better than 100 grams for total body mass), calibrated accuracy to four-compartment body composition models, and very low X-ray dose per exam (10 µSv or less), DXA is considered a gold standard for measuring body composition [17], [18], [19], [20], [21]. For these reasons, DXA systems have been used in many large-scale epidemiological studies to measure bone density and body composition. One of the largest national surveys to include DXA whole body scans was the Centers for Disease Control and Prevention’s National Health and Nutrition Examination Survey (NHANES); DXA was used to measure body composition in a representative sample of the United States population by gender, race/ethnicity, and age [22], [23], [24].

While ratios of DXA-reported fat masses have been used to distinguish certain groups, they are not as intuitive as body shape itself. We have recently developed a technique to measure solid body volume using DXA-reported fat, lean, and BMC values [25], [26]. DXA-based volume outperforms traditional air and water displacement techniques because regional body volumes can be measured and no assumptions are needed to correct for internal air voids. This method is powerful because it is applicable for retrospective analysis of large studies since it only requires access to standard DXA scan values.

In this study, we derived an easily interpretable body shape measure from whole body DXA data, the ratio of trunk volume to leg volume, and tested its association to diabetes, metabolic covariates, and subsequent mortality in a representative United States population. We hypothesized that participants with a higher trunk to leg volume ratio would have higher rates of diabetes, its metabolic covariates, and mortality.

## Methods

We performed a retrospective analysis of the publicly accessible NHANES 1999–2004 datasets to determine the association of body shape to diabetes, metabolic covariates, and mortality. NHANES is a population-based study of the non-institutionalized US population. In addition to DXA scan output, NHANES 1999–2004 contains self-reported survey responses (including gender, race/ethnicity, diabetes status, physical activity level, family size, family income level, various medication usage) and laboratory-based results (including weight, height, BMI, waist circumference, fasting plasma glucose, insulin, triglycerides, high-density lipoprotein (HDL), systolic blood pressure, and diastolic blood pressure). There were a total of 10,673 adult subjects (age > = 20 years) with DXA scan output data available from the public study website [27]. We excluded 797 individuals who had one of several quality-related issues due to either a non-removable artifact or body positioning in the DXA scan (e.g. missing limb, arm was off the scan table, metal implant, etc.) The total number of participants included in the final data analysis was 9876∶3120 individuals were from years 1999–2000, 3523 from years 2001–2003, and 3233 from years 2003–2004. Prospective mortality, coded as “Assumed alive” or “Assumed deceased”, was available for download on the NHANES public study website by a linkage with the National Death Index through 12/31/2006 [27].

We generated whole body and regional (arms, legs, and trunk) volume measures from the DXA scan output by using the calibration equation described in a previous reporting [25]: DXA_volume_ = Fat/0.88+ Lean/1.05+ BMC/4.85+0.01. We created our body shape index as the ratio of trunk volume to leg volume.

We analyzed the distribution of demographic variables by BMI category and tested for differences between these groups by the Bonferroni-adjusted t-test. We examined the prevalence of pre-diabetes (fasting plasma glucose levels between 100–125 mg/dL) and diabetes (defined by self- reported diagnosis or a fasting glucose ≥126 mg/dL) by gender, race/ethnicity, age category, BMI category, weight quartile, DXA total percent fat quartile, trunk to leg volume ratio quartile, waist circumference category, triglyceride level, HDL-cholesterol level, and blood pressure category. To avoid the confounding effect of medication use of individuals with diabetes on their lipids and blood pressure, we used quartile cut points derived from the population excluding those with diabetes. We used the 2005 NCEP guidelines to define cut points for BMI categories, high waist circumference, high triglyceride levels, high blood pressure, low HDL levels, and metabolic syndrome [28].For the metabolic syndrome definition, we only had enough information to determine whether individuals were taking insulin, diabetes pills, or antihypertension medication; information about fibrates or niacin was not available in this iteration of NHANES. We determined whether there was a significant trend in the prevalence of diabetes, high triglycerides, low HDL, and high blood pressure with trunk to leg volume quartile. To determine whether these trends differed by sub-group, we examined the distribution of individuals in each quartile of trunk to leg volume ratio with each outcome by BMI category, gender, and race/ethnicity group. Additionally, we investigated the prevalence of metabolic syndrome and mortality rate in each quartile of trunk to leg volume ratio by BMI category, gender, race/ethnicity group, and age category. We also determined whether there was significant interaction between trunk to leg volume ratio quartile and subgroup (BMI category, gender, race/ethnicity, and age category) for each outcome.

We used sequential logistic regression models to determine the association between trunk to leg volume ratio and metabolic outcomes (diabetes, high triglycerides, low HDL-cholesterol levels, high blood pressure, and metabolic syndrome) and mortality. For each model, we determined the order of variable significance, area under the receive-operator characteristic curve (AUC), odds ratio per standard deviation increase of trunk to leg volume ratio, and odds ratios for trunk to leg volume quartile (compared to the first quartile). We first adjusted for age alone; in the second stage covariate model, we also included gender, race/ethnicity, continuous BMI, continuous waist circumference, self-reported activity level, continuous poverty index ratio. To adjust for other DXA-derived measures of body fat, we created a second covariate model that also adjusted for the ratio of trunk fat mass to leg fat mass. The full model (used only for diabetes) included all covariates above and further adjusted for fasting insulin, triglycerides, HDL, systolic and diastolic blood pressure. We finally created a second full model (used only for diabetes) that also adjusted for the ratio of trunk fat mass to leg fat mass. All statistical analysis was done using SAS software, version 9.2 (SAS Institute, Inc., Cary, NC).

To investigate the driving forces of fat mass and lean mass behind trunk to leg volume ratio, we generated several height-normalized variables: trunk fat mass index (kg/m^2^), trunk lean mass index (kg/m^2^), trunk volume index (L/m^2^), leg fat mass index (kg/m^2^), leg lean mass index (kg/m^2^), and leg volume index (L/m^2^). To determine what body composition variables affected trunk to leg volume the most, we compared mean values of these height-normalized variables, trunk to leg fat mass ratio, and trunk to leg lean mass ratio to trunk to leg volume ratio quartile.

## Results

Table 1 shows that for most demographic variables, there were significant differences between BMI categories. There were not statistically significant differences for trunk to leg volume ratio between overweight/obese BMI groups, for triglycerides between overweight/obese and underweight/normal BMI groups, for systolic blood pressure between overweight/obese and underweight/normal BMI groups, and for diastolic blood pressure between underweight/normal and underweight/overweight BMI groups. Table 2 shows that the prevalence of both pre-diabetes and diabetes increased with age, BMI, weight, waist circumference, trunk to leg volume ratio, triglyceride level, blood pressure, and decreased HDL level.

**Table 1:** Demographics of individuals analyzed in NHANES 1999–2004 by BMI category displayed as total number (for gender and race/ethnicity) and mean ± standard deviation for all other measures.

Demographic	Underweight	Normal	Overweight	Obese	Total
Female	102	1645	1572	1496	4815
Male	67	1612	2251	1131	5061
Mexican American	17	627	994	699	2337
Non-Hispanic Black	35	520	647	569	1771
Non-Hispanic White	100	1799	1860	1193	4952
Other Race/Ethnicity	17	311	322	166	816
Age (yr)	43.7±20.2	47.1±19.6	51.4±17.9	49.4±16.7	49.3±18.3
BMI (kg/m^2^)	17.5±0.9	22.5±1.7	27.4±1.4	33.7±3.2	27.3±5.0
Weight (kg)	48.8±6.6	63.6±9.0	77.9±10.1	93.2±13.1	76.8±16.0
Waist Circumference (cm)	70.2±5.2	83.1±7.7	96.3±7.6	108.5±8.9	94.8±13.0
Triglycerides (mg/dL)a,b	96±64	117±87	160±151	173±182	148±144
HDL (mg/dL)	61.3±16.8	56.7±16.5	49.4±14.7	46.8±12.8	51.4±15.5
Systolic BP (mmHg)a,b	119±24	122±21	128±20	128±19	126±21
Diastolic BP (mmHg)a,c	70±12	69±13	72±13	73±13	71±13
DXA Total Fat (%)	22.9±6.2	29.0±7.7	33.5±7.3	39.8±7.2	33.5±8.6
Trunk to Leg Fat Mass Ratio^a^	1.04±0.31	1.30±0.43	1.61±0.47	1.63±0.45	1.50±0.48
Trunk to Leg Volume Ratio^a^	1.40±0.17	1.46±0.22	1.57±0.24	1.57±0.26	1.53±0.24

BMI categories were defined as follows: underweight BMI (<18.5 kg/m^2^), normal BMI (> = 18.5 kg/m^2^ and <25 kg/m^2^), overweight BMI (> = 25 kg/m^2^ and <30 kg/m^2^), and obese BMI (> = 30 kg/m^2^). All measures displayed were significantly different (P<0.05) for each BMI category (by Bonferroni-adjusted t-test) unless otherwise noted.

a Differences between Overweight & Obese were not significantly significant.

b Differences between Underweight & Normal were not significantly significant.

c Differences between Underweight & Overweight were not significantly significant.

**Table 2:** Prevalence of pre-diabetes and diabetes by selected measures in NHANES 1999–2004.

Measure	Value	N	% Pre-Diabetes	% Diabetes
Gender	Female	4815	10.0	10.2
	Male	5061	15.7	10.4
Race/Ethnicity	Mexican American	2337	14.9	13.7
	Non-Hispanic Black	1771	8.7	11.8
	Non-Hispanic White	4952	13.6	7.9
	Other	816	12.3	12.0
Age (yr) a,b	<50	5246	9.5	3.2
	50–70	2901	16.0	17.6
	>70	1729	18.1	19.5
BMI (kg/m^2^) a,b	Underweight (<18.5)	169	8.3	3.0
	Normal (18.5–25)	3257	9.4	6.0
	Overweight (25–30)	3823	15.2	11.4
	Obese (>30)	2627	14.3	14.5
Weight (kg) a,b	<64.8 kg (Q1)	2380	8.6	7.5
	≥64.8 kg & <75.2 kg (Q2)	2447	11.8	9.7
	≥75.2 kg & <86.7 kg (Q3)	2512	15.5	11.1
	≥86.7 kg (Q4)	2537	15.5	12.7
DXA Total Fat (%) b	<26.9% (Q1)	2334	11.4	6.2
	≥26.9% & <32.8% (Q2)	2466	15.2	10.6
	≥32.8% & <40.3% (Q3)	2517	12.7	11.3
	≥40.3(Q4)	2559	12.4	12.8
Trunk to Leg Volume Ratio a,b	<1.34 (Q1)	2282	6.1	2.9
	≥1.34 & <1.50 (Q2)	2339	10.0	5.3
	≥1.50 & <1.66 (Q3)	2401	15.6	7.8
	≥1.66 (Q4)	2854	18.5	22.4
Waist Circumference (cm)	≤102 (M) or ≤88 (F) (Low)	5257	14.7	15.1
	>102 (M) or >88 (F) (High)	4619	11.3	6.0
Triglycerides (mg/dL)	<150 (Low)	3089	23.8	8.0
	≥150 (High)	1578	33.5	19.8
HDL (mg/dL)	<40 (M) or <50 (F) (Low)	2207	15.7	14.5
	≥40 (M) or ≥50 (F) (High)	4114	13.0	7.4
Blood Pressure (mmHg)	<130 (S) & <85 (D) (Low)	5944	11.0	6.8
	≥130 (S) or ≥85 (D) (High)	3715	16.0	15.6

Quartile cut points (Q1–Q4) were based on individuals without diabetes. For waist circumference and HDL levels, there were separate cutoffs by gender, so ‘M’ is male and ‘F’ is female. Systolic blood pressure is shown as ‘S’, and diastolic blood pressure is shown as ‘D’.

a Pre-Diabetes P-for-trend <0.05.

b Diabetes P-for-trend <0.05.

**Figure 1:**
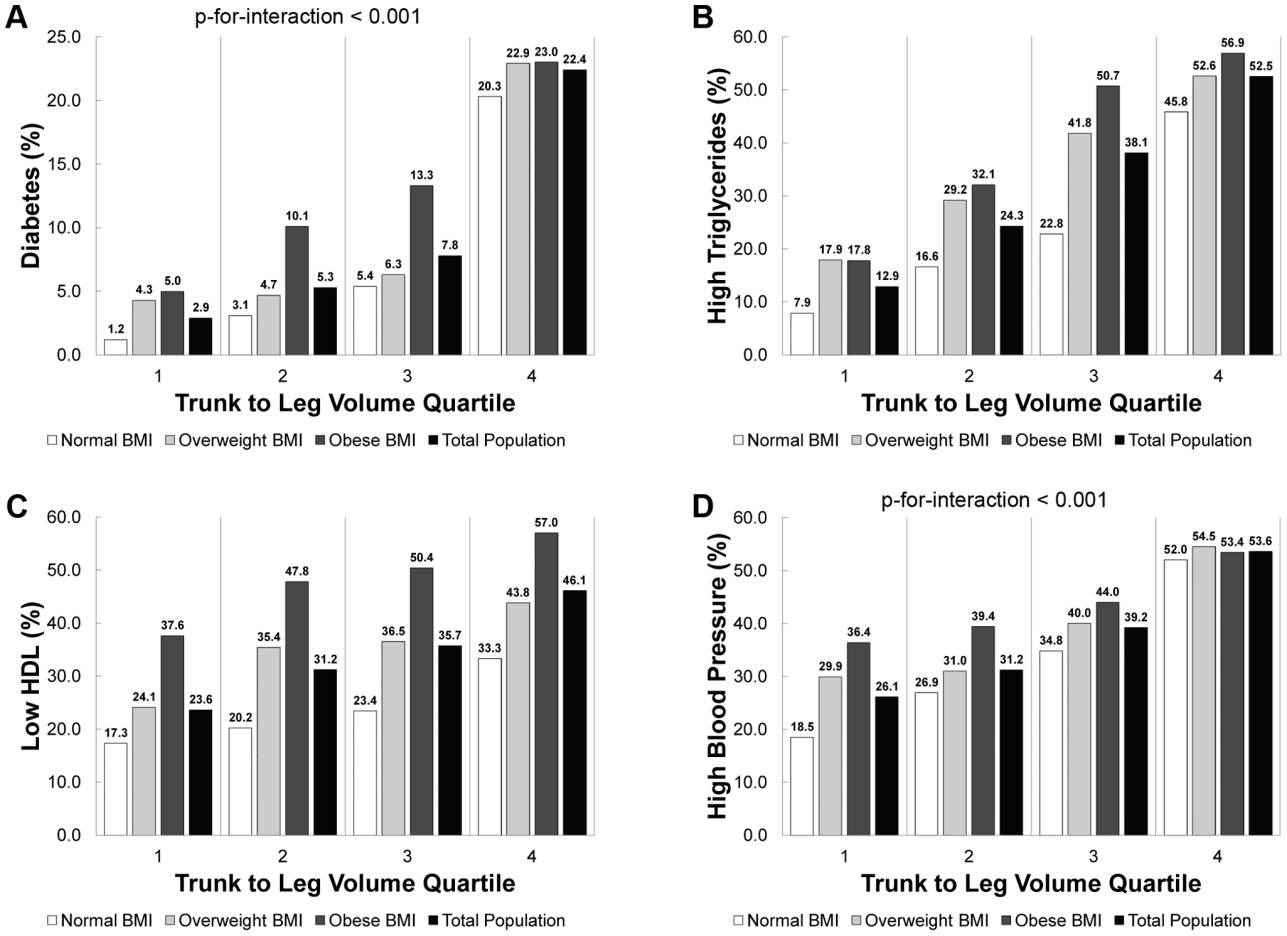
Prevalence of diabetes and metabolic covariates versus trunk to leg volume ratio by BMI category. The prevalence of diabetes (A), high triglycerides (B), low HDL (C), and high blood pressure (D) versus trunk to leg volume ratio quartile for normal BMI (> = 18.5 kg/m^2^ and <25 kg/m^2^), overweight BMI (> = 25 kg/m^2^ and <30 kg/m^2^), obese BMI (> = 30 kg/m^2^), and total population in NHANES 1999–2004 are shown below. All data displayed had a significant trend (P-for-trend <0.001) in prevalence versus quartile of trunk to leg volume ratio. There was a significant (P<0.001) interaction (trunk to leg volume ratio quartile & BMI category) in the prevalence of diabetes and high blood pressure.

Figure 1 shows that, in both the total population and those with normal BMI (> = 18.5 kg/m^2^ and <25 kg/m^2^), the prevalence of each outcome increased by trunk to leg volume ratio quartile (P-for-trend<0.001). There was a significant interaction (P-for-interaction<0.001) between trunk to leg volume ratio quartile and BMI category with the highest quartile of trunk to leg volume ratio having equally high diabetes and high blood pressure prevalence regardless of BMI category.

**Figure 2:**
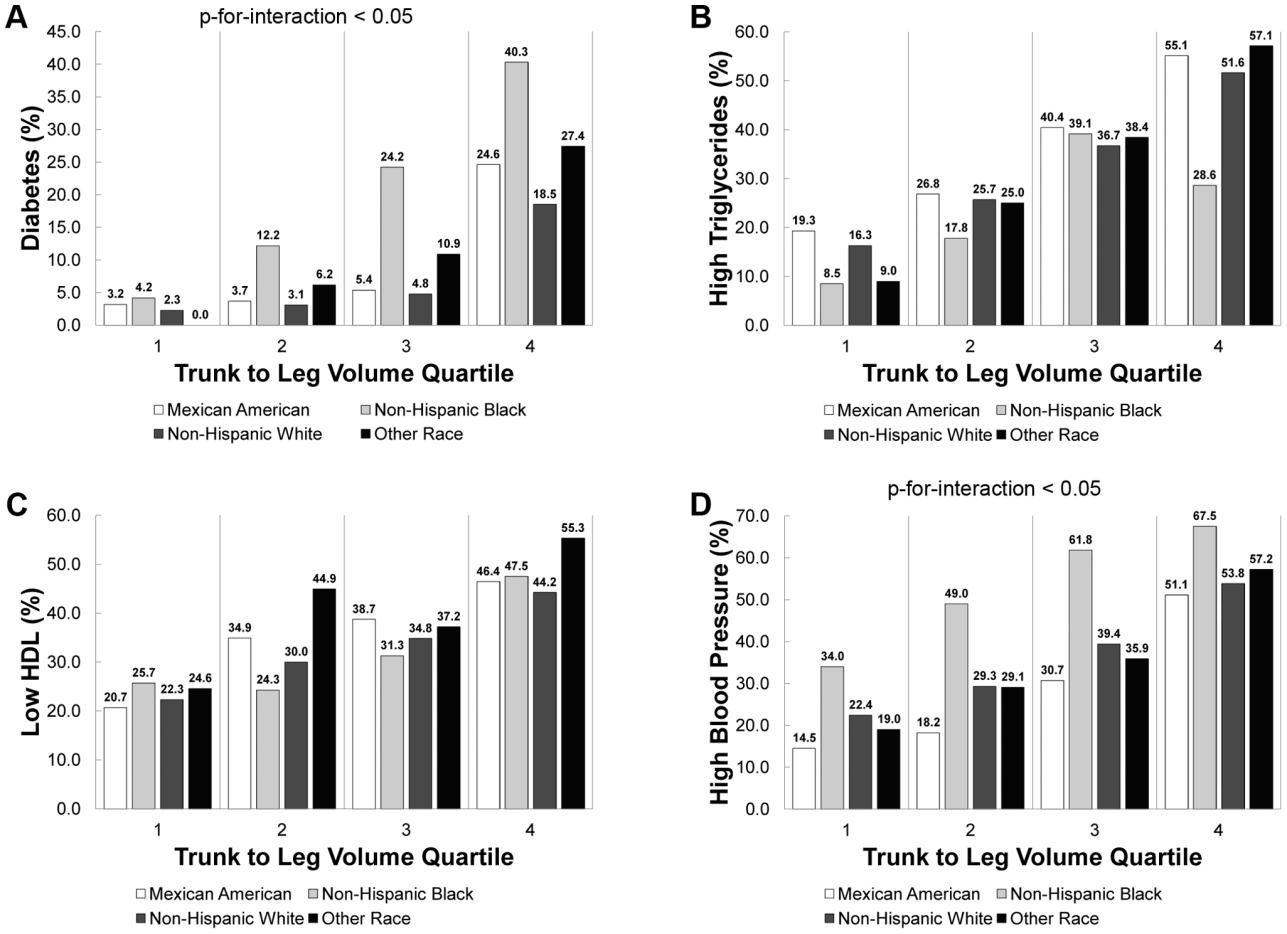
Prevalence of diabetes and metabolic covariates versus trunk to leg volume ratio quartile by race/ethnicity. The prevalence of diabetes (A), high triglycerides (B), low HDL (C), and high blood pressure (D) versus trunk to leg volume ratio quartile for race/ethnicity in NHANES 1999–2004 are shown below. All data displayed had a significant trend (P-for-trend <0.001) in prevalence versus quartile of trunk to leg volume ratio. There was a significant (P<0.05) interaction term (trunk to leg volume ratio quartile & race/ethnicity) in the prevalence of diabetes and high blood pressure.

Figure 2 shows that, for all race/ethnicity groups, the prevalence of diabetes, high triglycerides, low HDL, and high blood pressure increased as a function of trunk to leg volume ratio quartile (P-for-trend <0.0001). Again, there was a significant interaction (P-for-interaction<0.05) between trunk to leg volume ratio quartile and race/ethnicity group for diabetes and high blood pressure prevalence. Non-Hispanic Black individuals saw a relatively steady increase in diabetes prevalence by trunk to leg volume ratio quartile, while other race/ethnicity groups saw a dramatic increase in prevalence from the third to fourth quartile of trunk to leg volume ratio. Non-Hispanic Black individuals have the highest overall prevalence in high blood pressure for each quartile of trunk to leg volume ratio.

There was a significant increasing trend (P-for-trend<0.001) in the prevalence of diabetes, high triglycerides, low HDL, and high blood pressure by trunk to leg volume ratio quartile for both men and women.

**Figure 3:**
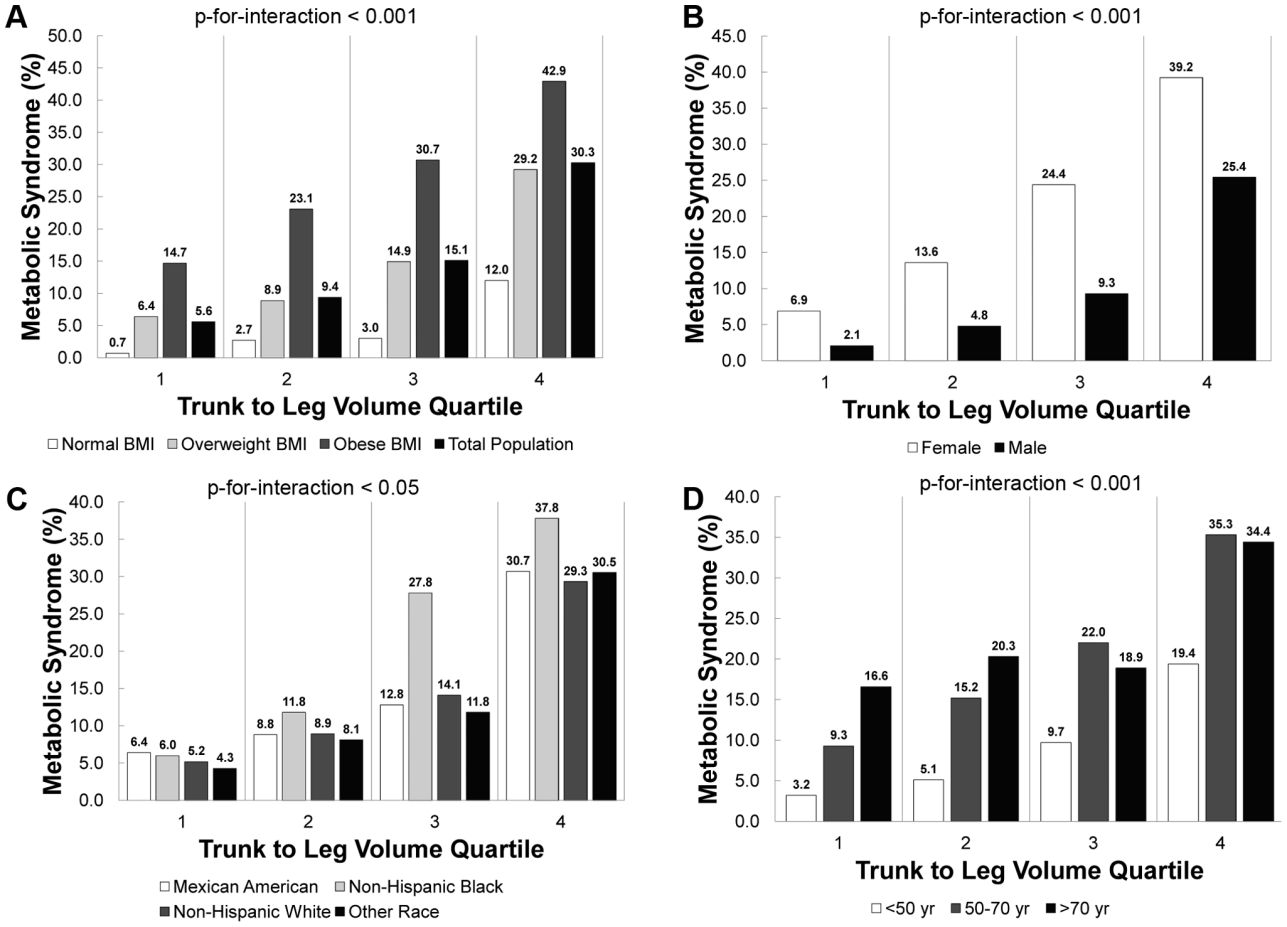
Prevalence of metabolic syndrome versus trunk to leg volume ratio quartile by BMI, gender, race/ethnicity, and age. The prevalence of metabolic syndrome versus trunk to leg volume ratio quartile is displayed below for (A) BMI category, (B) gender, (C) race/ethnicity, and (D) age group in NHANES 1999–2004. (A) All data displayed by BMI category had a significant trend (P-for-trend<0.001) in metabolic syndrome versus trunk to leg volume ratio quartile; there was also a significant interaction (P-for-interaction<0.001) between trunk to leg volume ratio quartile and BMI category for metabolic syndrome. (B) All data displayed by gender had a significant trend (P-for-trend <0.001) in mortality versus trunk to leg volume ratio quartile; there was also a significant interaction (P-for-interaction<0.001) between trunk to leg volume ratio quartile and gender for metabolic syndrome. (C) All data displayed by race/ethnicity had a significant trend (P-for-trend<0.001) in metabolic syndrome versus trunk to leg volume ratio quartile; there was also a significant interaction (P-for-interaction<0.05) between trunk to leg volume ratio quartile and race/ethnicity for metabolic syndrome. (D) All data displayed by age group had a significant trend (P-for-trend<0.001) in metabolic syndrome versus trunk to leg volume ratio quartile; there was also a significant interaction (P-for-interaction<0.001) between trunk to leg volume ratio quartile and age group for metabolic syndrome.

Figure 3 shows that, for each BMI category, gender, race/ethnicity group, and age group, the prevalence of metabolic syndrome increased by trunk to leg volume ratio quartile (P-for-trend<0.001). The effect of trunk to leg volume ratio varied by BMI category (P-for-interaction<0.001), gender (P-for-interaction<0.001), race/ethnicity group (P-for-interaction<0.05), and age group (P-for-interaction<0.001). While those with obese BMI had the highest prevalence of metabolic syndrome, those with normal BMI had the largest increase in metabolic syndrome prevalence from the third to fourth quartile of trunk to leg volume ratio (3.0% to 12.0%). While women had a higher prevalence of metabolic syndrome across all quartiles, men had a large jump from the third to fourth quartile (9.3% to 25.4%). Non-Hispanic Black individuals had the highest prevalence of metabolic syndrome for almost all quartiles of trunk to leg volume ratio, but Non-Hispanic White and Other Race individuals had major increases in prevalence from third to fourth quartiles (14.1% to 29.3% and 11.8% to 30.5%, respectively). While individuals over 70 years and between 50 and 70 years had consistently a consistently higher prevalence of metabolic syndrome across all quartiles of trunk to leg volume ratio, individuals under 50 years had the largest increase in prevalence from the third to fourth quartiles (9.7% to 19.4%).

**Figure 4:**
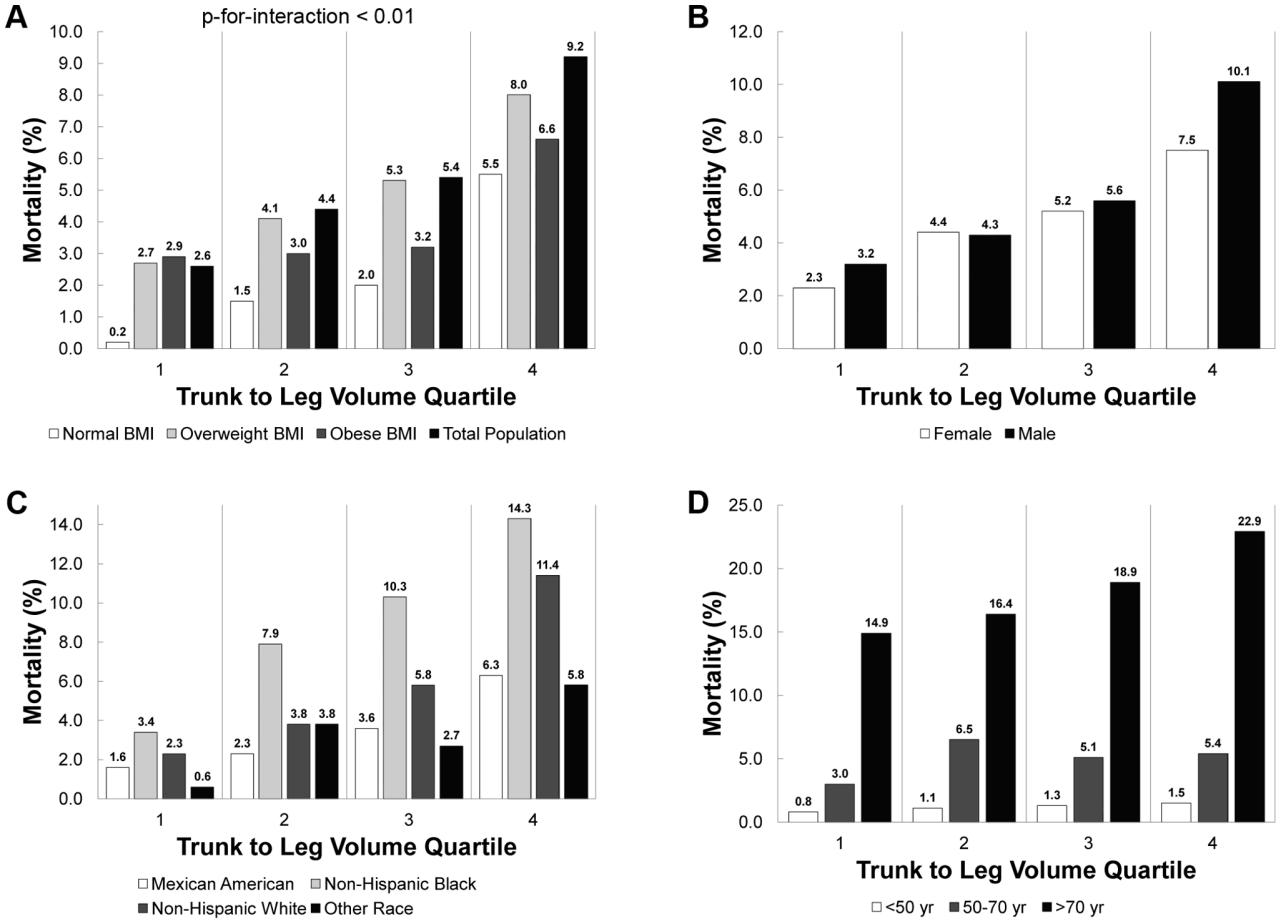
Mortality versus trunk to leg volume ratio quartile by BMI, gender, race/ethnicity, and age. Mortality versus trunk to leg volume ratio quartile is displayed below for (A) BMI category, (B) gender, (C) race/ethnicity, and (D) age group in NHANES 1999–2004. (A) All data displayed by BMI category had a significant trend (P-for-trend<0.001) in mortality versus trunk to leg volume ratio quartile; there was also a significant interaction (P-for-interaction<0.01) between trunk to leg volume ratio quartile and BMI category for mortality. (B) All data displayed by gender had a significant trend (P-for-trend <0.001) in mortality versus trunk to leg volume ratio quartile. (C) All data displayed by race/ethnicity had a significant trend (P-for-trend<0.001 for Mexican American, Non-Hispanic Black, and Non-Hispanic White; P-for-trend<0.05 for Other Race)) in mortality versus trunk to leg volume ratio quartile. (D) Only individuals >70 years displayed a significant trend (P-for-trend <0.01) in mortality versus trunk to leg volume ratio quartile.

Figure 4 shows that, for each BMI category, gender, and race/ethnicity group, mortality increased by trunk to leg volume ratio quartile (P-for-trend<0.001 except “Other Race” which had a P-for-trend<0.05). In an analysis of mortality by age group, we found only those individuals over 70 years had a significant increasing trend (P-for-trend <0.01) by trunk to leg volume ratio quartile. Overall, mortality increased as a function of trunk to leg volume ratio quartile in the total population, within BMI categories, within gender groups, within race/ethnicity groups, and for those over 70 years old. The effect of trunk to leg volume ratio varied by BMI category for mortality (P-for-interaction<0.01). Those with overweight BMI had a higher prevalence of mortality with increasing quartile of trunk to leg volume ratio than for other BMI categories.

Table 3 shows that, even after adjusting for confounders, a high trunk to leg volume ratio was still associated with diabetes, high triglycerides, low HDL, high blood pressure, metabolic syndrome, and subsequent mortality. In the covariate logistic regression model, we found that individuals in the highest quartile of trunk to leg volume ratio had increased odds of having diabetes (Odds Ratio [OR] = 6.8, 95% confidence interval [CI] 4.9–9.6), high triglycerides (OR = 3.9, 95% CI 3.0–5.2), low HDL (OR = 3.0, 95% CI 2.4–3.8), and high blood pressure (OR = 1.8, 95% CI 1.6–2.1) compared to those in the lowest quartile. Even after adjusting for DXA-derived trunk to leg fat mass ratio in the Covariate 2 model, we found that individuals in the highest quartile of trunk to leg volume ratio had increased odds of having diabetes (OR = 2.6, 95% CI 1.7–4.0), high blood pressure (OR = 1.4, 95% CI 1.1–1.8), metabolic syndrome (OR = 1.6, 95% CI 1.1–2.3), and mortality (OR = 1.8, 95% CI 1.2–2.6).

**Table 3:** Results of logistic regression models to distinguish those individuals with diabetes, high triglycerides (TG), low HDL, high blood pressure (BP), metabolic syndrome (MetS), and mortality in NHANES 1999–2004 by trunk to leg volume ratio.

			Odds Ratios for Trunk to Leg Volume Ratio Quartile				
Condition	Model	AUC	Per SD Increase	Q1	Q2	Q3	Q4
Diabetes	Age^a^	0.796	2.2 (2.0–2.3)	1.0	1.6 (1.2–2.2)	2.1 (1.6–2.8)	5.7 (4.4–7.4)
	Covariate^b^	0.839	2.3 (2.1–2.5)	1.0	2.0 (1.4–2.8)	2.6 (1.9–3.7)	6.8 (4.9–9.6)
	Covariate 2^c^	0.839	2.3 (2.1–2.5)	1.0	1.6 (1.1–2.2)	1.6 (1.1–2.3)	2.6 (1.7–4.0)
	Full^d^	0.868	1.9 (1.6–2.3)	1.0	1.1 (0.6–2.0)*	1.4 (0.8–2.5)*	3.9 (2.2–7.0)
	Full 2^e^	0.868	1.9 (1.6–2.3)	1.0	0.9 (0.5–1.8)*	1.1 (0.6–2.0)*	2.2 (1.0–4.7)
High TG	Age	0.703	2.1 (1.9–2.2)	1.0	2.1 (1.7–2.7)	4.0 (3.2–5.0)	6.8 (5.5–8.5)
	Covariate	0.722	1.8 (1.6–1.9)	1.0	1.7 (1.4–2.2)	2.8 (2.2–3.7)	3.9 (3.0–5.2)
	Covariate 2	0.538	0.8 (0.7–0.9)	1.0	0.9 (0.8–1.1)*	0.8 (0.7–1.0)	0.7 (0.6–0.9)
Low HDL	Age	0.628	1.7 (1.6–1.8)	1.0	1.6 (1.3–1.8)	2.1 (1.8–2.4)	3.6 (3.0–4.2)
	Covariate	0.705	1.6 (1.5–1.7)	1.0	1.5 (1.2–1.8)	2.0 (1.6–2.4)	3.0 (2.4–3.8)
	Covariate 2†	0.709	1.2 (0.7–2.4)*	1.0	1.2 (1.0–1.5)*	1.2 (1.0–1.5)*	1.3 (0.9–1.7)*
High BP	Age	0.768	1.2 (1.1–1.3)	1.0	1.1 (0.9–1.3)*	1.3 (1.1–1.4)*	1.6 (1.4–1.8)
	Covariate	0.781	1.3 (1.2–1.3)	1.0	1.2 (1.0–1.4)	1.4 (1.2–1.7)	1.8 (1.6–2.1)
	Covariate 2†	0.782	1.9 (1.1–3.2)	1.0	1.1 (1.0–1.4)*	1.2 (1.0–1.5)	1.4 (1.1–1.8)
MetS	Age	0.747	1.9 (1.8–2.1)	1.0	1.6 (1.3–2.0)	2.5 (2.0–3.0)	4.9 (4.0–6.1)
	Covariate	0.840	1.8 (1.6–1.9)	1.0	1.5 (1.2–2.0)	2.2 (1.7–2.9)	3.6 (2.8–4.7)
	Covariate 2†	0.840	1.4 (1.2–1.6)	1.0	1.2 (1.0–1.6)*	1.5 (1.1–2.0)	1.6 (1.1–2.3)
Mortality	Age	0.830	1.2 (1.1–1.3)	1.0	1.4 (1.0–2.0)	1.4 (1.1–2.0)	1.7 (1.2–2.3)
	Covariate	0.862	1.2 (1.1–1.4)	1.0	1.3 (0.9–1.9)*	1.4 (1.0–2.1)*	1.8 (1.2–2.6)
	Covariate 2	0.863	1.8 (1.5–2.3)	1.0	1.3 (0.9–1.9)*	1.4 (1.0–2.1)*	1.8 (1.2–2.6)

AUC is the area under the receiver-operator characteristic curve. Odds ratios are displayed as odds ratio (95% confidence interval). Quartile cut points (Q1-Q4) were based on individuals without diabetes.

a Age model adjusts for age.

b Covariate model adjusts for gender, race/ethnicity, age, BMI, waist circumference, self-reported activity level, and poverty index ratio.

c Covariate 2 model adjusts for gender, race/ethnicity, age, BMI, waist circumference, self-reported activity level, poverty index ratio, and trunk to leg fat mass ratio.

d Full model adjusts for all variables in ^b^ and insulin, triglycerides, HDL, systolic blood pressure, and diastolic blood pressure.

e Full 2 model adjusts for all variables in ^d^ and trunk to leg fat mass ratio.

* Odds ratio not significant.

† Forward selection turned off because trunk to leg volume ratio quartile wasn’t significant enough to remain in the model otherwise.

In a fully adjusted model, individuals in the highest quartile of trunk to leg volume ratio were 3.9 times as likely to have diabetes compared to the lowest quartile, but the odds of diabetes in the second or third quartiles were not significantly different than the lowest quartile. Additionally, trunk to leg volume ratio was the most significant variable, followed by age, in the fully-adjusted model (P<0.001). Even after adjusting for DXA-derived trunk to leg fat mass ratio in the Full 2 model, we found that individuals in the highest quartile of trunk to leg volume ratio were 2.2 times as likely to have diabetes compared to the lowest quartile.

For mortality, the association with waist circumference was not significant. Individuals in the highest quartile of trunk to leg volume ratio had increased odds of mortality (OR = 1.8, 95% CI 1.2–2.6) compared to those in the lowest quartile while there was a decreased odds of mortality with each SD increase in BMI (OR = 0.7, 95% CI 0.7–0.8). For several of the models that also adjusted for trunk to leg fat mass ratio (Covariate 2), forward selection of variables was turned off to ensure that both trunk to leg fat mass ratio and trunk to leg volume ratio remained in the model.

**Figure 5:**
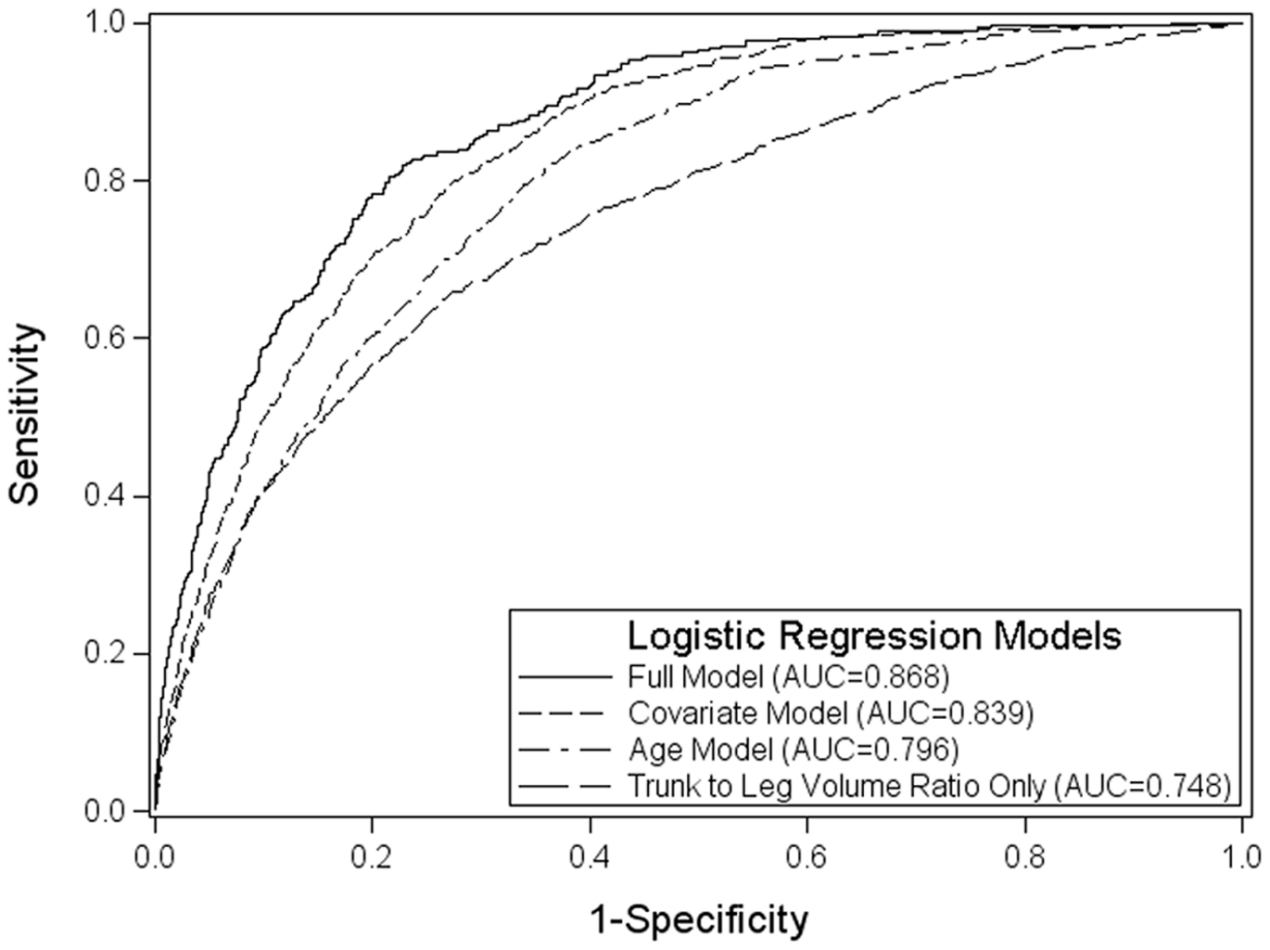
Diabetes Receiver Operating Characteristic (ROC) for Logistic Regression Models in NHANES 1999–2004. Each ROC curve displays the sensitivity versus one minus specificity for each logistic regression model that is used to distinguish those individuals with diabetes in NHANES 1999–2004. The trunk to leg volume ratio only model (AUC = 0.748) includes only the variable of trunk to leg volume ratio. The age model (AUC = 0.796) includes the variables of age and trunk to leg volume ratio. The covariate model (AUC = 0.839) includes the variables of gender, race/ethnicity, age, BMI, waist circumference, self-reported activity level, poverty index ratio, and trunk to leg volume ratio. The full model (AUC = 0.796) includes the variables of race/ethnicity, age, waist circumference, poverty index ratio, insulin, triglycerides, systolic blood pressure, diastolic blood pressure, and trunk to leg volume ratio; gender, BMI, self-reported activity level, and HDL level were dropped from the final model because the coefficients were not significant (P<0.05).

Figure 5 displays the receiver operator characteristics (ROC) curves for four progressively more complex models to distinguish those individuals with diabetes. Using forward logistic regression with all significant variables, we found that trunk to leg volume ratio was the variable that contributed the most to distinguish those with diabetes. Using only truck to leg volume ratio, we found that the area under the ROC curve (AUC) was 0.748. Adding the second most contributing variable, age, increased the AUC to 0.796. The covariate model increased the AUC to 0.839, and finally the full model had an AUC of 0.868.

**Figure 6:**
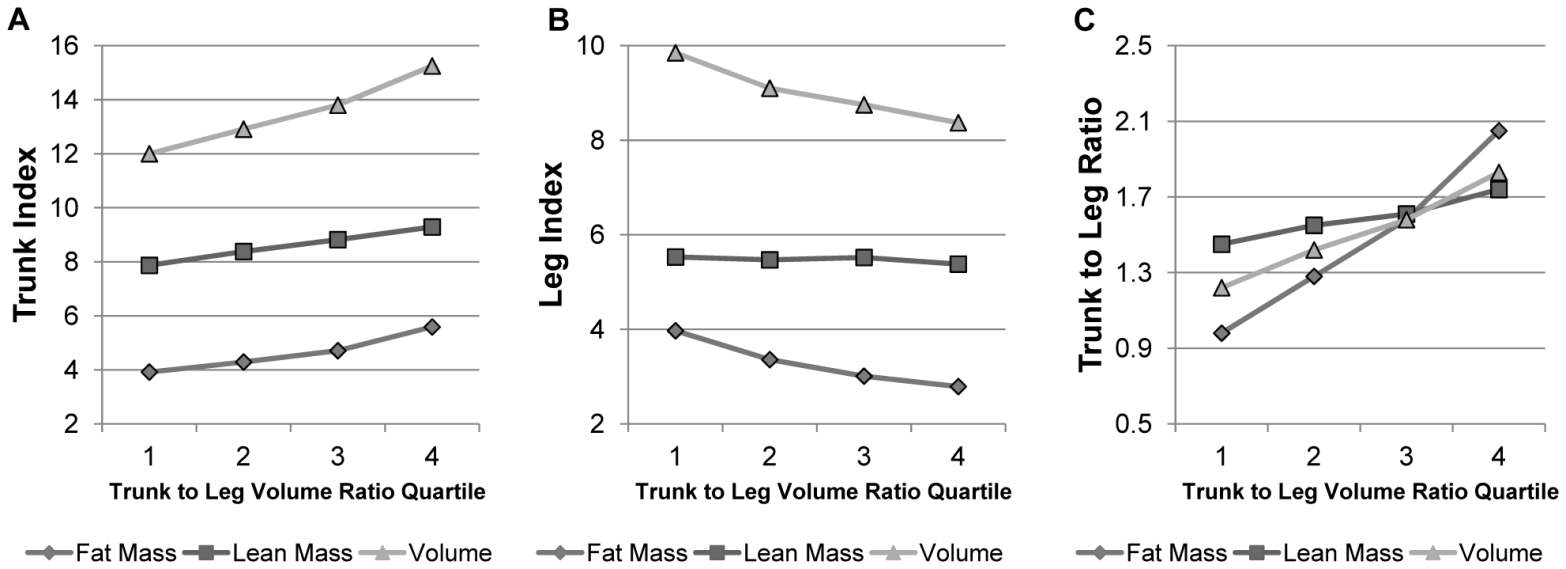
Breakdown of trunk to leg volume ratio by its major components. (A) Mean height-normalized trunk fat mass index (kg/m^2^), trunk lean mass index (kg/m^2^), and trunk volume index (L/m^2^) values are stratified by quartile of trunk to leg volume ratio. The increase in trunk volume is attributed mainly to the increase in trunk fat. (B) Mean height-normalized leg fat mass index (kg/m^2^), leg lean mass index (kg/m^2^), and leg volume index (L/m^2^) values are stratified by quartile of trunk to leg volume ratio. There is an overall decrease in leg volume primarily driven by a decrease in leg fat mass. (C) Mean trunk to leg fat mass ratio, trunk to leg lean mass ratio, and trunk to leg volume ratio are stratified by quartile of trunk to leg volume ratio. Trunk to leg fat mass ratio increases more dramatically than trunk to leg lean mass ratio.

Figure 6 displays the behavior of height-normalized fat mass, lean mass, and volume in the trunk and legs as a function of trunk to leg volume ratio quartile. The increase in trunk volume is primarily driven by an increase in trunk fat, while the decrease in leg volume is primarily driven by a decrease in leg fat. Trunk to leg fat mass ratio has the steepest increase because of its increase in trunk fat mass and decrease in leg fat mass. Trunk to leg lean mass ratio has a more attenuated increase because of its shallower increase in trunk lean mass and stable leg lean mass values as a trunk to leg volume ratio increases. Increases in total trunk fat were most likely driven by increases in visceral fat that overwhelmed the decreases in overall subcutaneous fat represented by the loss of fat mass in the legs, but this study was unable to isolate visceral from subcutaneous fat.

## Discussion

The prevalence of diabetes, high triglycerides, low HDL, high blood pressure, metabolic syndrome, and subsequent mortality significantly increased with each trunk to leg volume ratio quartile. Among traditionally low-risk individuals in the normal BMI category, prevalence of these conditions increased dramatically as trunk to leg volume ratio increased. Even after adjusting for several covariates in the pathway between body shape and the metabolic outcome including the fat distribution measure of trunk to leg fat mass ratio, individuals in the fourth versus first quartile of trunk to leg volume ratio had significantly increased odds of having diabetes, high triglycerides, low HDL, high blood pressure, metabolic syndrome, and subsequent mortality. Additionally, the driving force behind increased trunk to leg volume ratio was primarily increases in both the fat and lean compartments of the trunk with decreases in the legs. Even after adjustments for other measures of body shape (BMI or waist circumference), trunk to leg volume ratio was an independent marker of diabetes, metabolic covariates, and mortality in a representative sample of the United States.

While simplistic shape measures of BMI and waist circumference are associated to diabetes status, our results show that trunk to leg volume ratio provides additional information beyond these measures. Most studies use BMI and waist circumference as surrogates for total percent fat and central adiposity, respectively [29], [30], [31], [32]. In 2012, Krakauer and Krakauer developed a new body shape index that predicted mortality hazard independently of BMI in NHANES 1999–2004 by adjusting waist circumference by height and BMI [33]. While these tools are powerful clinically because they are inexpensive and easy to measure, they do not represent body composition and shape accurately [34], [35], [36], [37]. In 2012, the US Preventative Services Task Force recommended that clinicians screen for obesity but recognized that the specific mechanism for screening needed additional research [38].

Because of the compounding effects of obesity and diabetes, trunk to leg volume ratio could potentially be used as a screening assessment that seems more promising than BMI or waist circumference. Mokdad et al. reported similar trends in diabetes prevalence by age and BMI category in the 2001 Behavioral Risk Factor Surveillance System [3]. Novotny, et al, previously reported that two surrogates of body shape (DXA-reported trunk to peripheral fat mass ratio and DXA-reported android to gynoid fat mass ratio) were significantly different between Asian and White adolescents [15], [16]. However, we recently reported that, in a repeat-measure subset of the NHANES 1999–2004 study, our measures of trunk to leg volume ratio and trunk to peripheral (arms and legs) volume ratio had better repeat-measure precision than trunk to leg fat mass ratio and trunk to peripheral fat mass ratio (3.27% and 3.09% versus 4.95% and 4.62%, respectively) [25].

Our study used DXA as a tool of convenience because of the availability of a large dataset for retrospective analysis. Because all DXA scans in NHANES 1999–2004 were taken on the same type of DXA system, no cross calibration between systems was necessary. While this project looks at regional DXA measures from the trunk and leg, we have previously developed a method to look at DXA-derived body shape on pixel-by-pixel basis that could be employed to generate advanced measures of shape beyond ratios of regional volume [39]. Ultimately, cheaper and potentially more accessible, optical methods could be used to measure body shape and assess risk for diabetes [37], [40].

Despite no statistically significant difference in mean trunk to leg volume ratio between overweight and obese individuals (in Table 1), we found that those individuals in the highest quartile of trunk to leg volume ratio had higher prevalence of all outcomes including mortality regardless of BMI category (Figure 1, 3, and 4). These data confirm that our measure of body shape adds more distinguishing power than BMI for many metabolic outcomes and mortality. The fully adjusted models for diabetes adjusted for potential mediators of adiposity, diabetes, and fat distribution; hence, the odds of having diabetes in the second and third (compared to the first) quartiles of trunk to leg volume ratio were attenuated significantly. Despite these major adjustments in the full models, the odds of having diabetes in the fourth (versus first) quartile of trunk to leg volume ratio remained highly significant (OR 3.9 and 2.2) and these models had the highest AUC.

Our initial hypothesis held true. Increased trunk to leg volume ratio was due to competing effects of adiposity and lean mass in the trunk and legs. We also hypothesized that increased trunk volume was due primarily to increased central adiposity, and that decreased leg volume was due to muscle wasting. However, we did not see changes in leg lean mass driving the trunk to leg volume ratio. Our data suggests that high visceral mass for both fat and lean accompanied with a low subcutaneous adiposity is the strongest driver of body shape risk irrespective of muscularity represented by leg lean mass.

Our study had several limitations. NHANES 1999–2004 didn’t include hip circumference measurements, so we were not able to do a direct comparison of trunk to leg volume ratio to waist to hip ratio, a surrogate of body shape used more in research than in clinical care. To compensate, we looked at the most similar measure we could generate (waist to thigh circumference ratio); this ratio was highly correlated to but did not perform as well as trunk to leg volume ratio. In 2012, two major DXA system manufacturers (Hologic and GE-Lunar) released feature updates to quantify visceral fat from their DXA scans [41], [42]. In future studies, we hope to further investigate the specific roles of visceral and subcutaneous fat using DXA-derived visceral fat measurements. Ultimately, our results were derived from prevalent diabetes and limited mortality data (through December 31, 2006) and need to be validated with more incident data to assess risk for developing diabetes and its metabolic covariates.

We conclude that this novel trunk to leg volume ratio derived from whole body DXA scans in a representative sample of the US population showed strong associations with diabetes, high triglycerides, low HDL, high blood pressure, metabolic syndrome and mortality. These associations were also strong for individuals in the normal BMI category, which is typically considered low risk for diabetes. Trunk to leg volume ratio provides an independent marker that intuitively describes body shape and stratifies diabetes and mortality more accurately than currently available body shape measures of BMI and waist circumference. A large ratio of trunk versus leg volume is a strong indicator of poor health, with increased prevalence of diabetes, poor metabolic profiles, and elevated mortality even in individuals not considered overweight.

## References

[1] Haffner SM, Lehto S, Ronnemaa T, Pyorala K, Laakso M (1998) Mortality from coronary heart disease in subjects with type 2 diabetes and in nondiabetic subjects with and without prior myocardial infarction. N Engl J Med 339: 229–234. 9673301 10.1056/NEJM199807233390404

[2] Hogan P, Dall T, Nikolov P (2003) Economic costs of diabetes in the US in 2002. Diabetes Care 26: 917–932. 12610059 10.2337/diacare.26.3.917

[3] Mokdad AH, Ford ES, Bowman BA, Dietz WH, Vinicor F, et al (2003) Prevalence of obesity, diabetes, and obesity-related health risk factors, 2001. Jama 289: 76–79. 12503980 10.1001/jama.289.1.76

[4] Deurenberg P, Weststrate JA, Seidell JC (1991) Body mass index as a measure of body fatness: age- and sex-specific prediction formulas. Br J Nutr 65: 105–114. 2043597 10.1079/bjn19910073

[5] Daniels SR, Khoury PR, Morrison JA (1997) The utility of body mass index as a measure of body fatness in children and adolescents: differences by race and gender. Pediatrics 99: 804–807. 9164773 10.1542/peds.99.6.804

[6] Pietrobelli A, Faith MS, Allison DB, Gallagher D, Chiumello G, et al (1998) Body mass index as a measure of adiposity among children and adolescents: a validation study. J Pediatr 132: 204–210. 9506629 10.1016/s0022-3476(98)70433-0

[7] Carey VJ, Walters EE, Colditz GA, Solomon CG, Willett WC, et al (1997) Body fat distribution and risk of non-insulin-dependent diabetes mellitus in women. The Nurses’ Health Study. Am J Epidemiol 145: 614–619. 9098178 10.1093/oxfordjournals.aje.a009158

[8] Wang Y, Rimm EB, Stampfer MJ, Willett WC, Hu FB (2005) Comparison of abdominal adiposity and overall obesity in predicting risk of type 2 diabetes among men. Am J Clin Nutr 81: 555–563. 15755822 10.1093/ajcn/81.3.555

[9] Carnethon MR, De Chavez PJ, Biggs ML, Lewis CE, Pankow JS, et al (2012) Association of weight status with mortality in adults with incident diabetes. Jama 308: 581–590. 22871870 10.1001/jama.2012.9282 PMC3467944

[10] Withers RT, LaForgia J, Pillans RK, Shipp NJ, Chatterton BE, et al (1998) Comparisons of two-, three-, and four-compartment models of body composition analysis in men and women. J Appl Physiol 85: 238–245. 9655781 10.1152/jappl.1998.85.1.238

[11] Behnke AR Jr, Feen BG, Welham WC (1942) The specific gravity of healthy men. Body weight divided by volume as an index of obesity. JAMA 118: 495–498. 10.1002/j.1550-8528.1995.tb00152.x 7627779

[12] Siri WE (1956) The gross composition of the body. Adv Biol Med Phys 4: 239–280. 13354513 10.1016/b978-1-4832-3110-5.50011-x

[13] Brozek J, Grande F, Anderson JT, Keys A (1963) Densitometric Analysis of Body Composition: Revision of Some Quantitative Assumptions. Ann N Y Acad Sci 110: 113–140. 14062375 10.1111/j.1749-6632.1963.tb17079.x

[14] Goldman RF, Buskirk ER (1959) Body Volume Measurement By Underwater Weighing: Description Of A Method. In: Brozek J, Henschel, A., editor; 1959 January 22; Natick, MA. National Academy of Sciences.

[15] Novotny R, Daida YG, Grove JS, Le Marchand L, Vijayadeva V (2006) Asian adolescents have a higher trunk:peripheral fat ratio than Whites. J Nutr 136: 642–647. 16484537 10.1093/jn/136.3.642 PMC1478165

[16] Novotny R, Going S, Teegarden D, Van Loan M, McCabe G, et al (2007) Hispanic and Asian pubertal girls have higher android/gynoid fat ratio than whites. Obesity (Silver Spring) 15: 1565–1570. 17557994 10.1038/oby.2007.185

[17] Ellis KJ, Shypailo RS, Steinberg FM, Lewis RD, Young RL, et al (2004) Reproducibility of fan-beam DXA measurements in adults and phantoms. J Clin Densitom 7: 413–418. 15618602 10.1385/jcd:7:4:413

[18] Hagiwara S, Lane N, Engelke K, Sebastian A, Kimmel DB, et al (1993) Precision and accuracy for rat whole body and femur bone mineral determination with dual X-ray absorptiometry. Bone Miner 22: 57–68. 8219938 10.1016/s0169-6009(08)80081-5

[19] Leonard CM, Roza MA, Barr RD, Webber CE (2009) Reproducibility of DXA measurements of bone mineral density and body composition in children. Pediatr Radiol 39: 148–154. 19052738 10.1007/s00247-008-1067-7

[20] Schoeller DA, Tylavsky FA, Baer DJ, Chumlea WC, Earthman CP, et al (2005) QDR 4500A dual-energy X-ray absorptiometer underestimates fat mass in comparison with criterion methods in adults. Am J Clin Nutr 81: 1018–1025. 15883424 10.1093/ajcn/81.5.1018

[21] Blake GM, Naeem M, Boutros M (2006) Comparison of effective dose to children and adults from dual X-ray absorptiometry examinations. Bone 38: 935–942. 16376161 10.1016/j.bone.2005.11.007

[22] Flegal KM, Ogden CL, Yanovski JA, Freedman DS, Shepherd JA, et al (2010) High adiposity and high body mass index-for-age in US children and adolescents overall and by race-ethnic group. Am J Clin Nutr 91: 1020–1026. 20164313 10.3945/ajcn.2009.28589 PMC2844683

[23] Flegal KM, Shepherd JA, Looker AC, Graubard BI, Borrud LG, et al (2009) Comparisons of percentage body fat, body mass index, waist circumference, and waist-stature ratio in adults. Am J Clin Nutr 89: 500–508. 19116329 10.3945/ajcn.2008.26847 PMC2647766

[24] Kelly TL, Wilson KE, Heymsfield SB (2009) Dual energy X-Ray absorptiometry body composition reference values from NHANES. PLoS One 4: e7038. 19753111 10.1371/journal.pone.0007038 PMC2737140

[25] Wilson JP, Fan B, Shepherd JA (2013) Total and Regional Body Volumes Derived From Dual-Energy X-Ray Absorptiometry Output. J Clin Densitom. 10.1016/j.jocd.2012.11.001 23321490

[26] Wilson JP, Strauss BJ, Fan B, Duewer FW, Shepherd JA (2013) Improved 4-compartment body-composition model for a clinically accessible measure of total body protein. Am J Clin Nutr. 10.3945/ajcn.112.048074 23364008

[27] CDC (2012) NHANES - National Health and Nutrition Examination Survey Centers for Disease Control and Prevention. Available: http://www.cdc.gov/nchs/nhanes.htm. Accessed 2013 Jan 5.

[28] Grundy SM, Cleeman JI, Daniels SR, Donato KA, Eckel RH, et al (2005) Diagnosis and management of the metabolic syndrome: an American Heart Association/National Heart, Lung, and Blood Institute Scientific Statement. Circulation 112: 2735–2752. 16157765 10.1161/CIRCULATIONAHA.105.169404

[29] Zhang C, Rexrode KM, van Dam RM, Li TY, Hu FB (2008) Abdominal obesity and the risk of all-cause, cardiovascular, and cancer mortality: sixteen years of follow-up in US women. Circulation 117: 1658–1667. 18362231 10.1161/CIRCULATIONAHA.107.739714

[30] Janssen I, Katzmarzyk PT, Ross R (2004) Waist circumference and not body mass index explains obesity-related health risk. Am J Clin Nutr 79: 379–384. 14985210 10.1093/ajcn/79.3.379

[31] Coutinho T, Goel K, Correa de Sa D, Kragelund C, Kanaya AM, et al (2011) Central obesity and survival in subjects with coronary artery disease: a systematic review of the literature and collaborative analysis with individual subject data. J Am Coll Cardiol 57: 1877–1886. 21545944 10.1016/j.jacc.2010.11.058

[32] de Koning L, Merchant AT, Pogue J, Anand SS (2007) Waist circumference and waist-to-hip ratio as predictors of cardiovascular events: meta-regression analysis of prospective studies. Eur Heart J 28: 850–856. 17403720 10.1093/eurheartj/ehm026

[33] Krakauer NY, Krakauer JC (2012) A new body shape index predicts mortality hazard independently of body mass index. PLoS One 7: e39504. 22815707 10.1371/journal.pone.0039504 PMC3399847

[34] Fujimoto WY, Bergstrom RW, Boyko EJ, Chen KW, Leonetti DL, et al (1999) Visceral adiposity and incident coronary heart disease in Japanese-American men. The 10-year follow-up results of the Seattle Japanese-American Community Diabetes Study. Diabetes Care 22: 1808–1812. 10546012 10.2337/diacare.22.11.1808

[35] Nicklas BJ, Cesari M, Penninx BW, Kritchevsky SB, Ding J, et al (2006) Abdominal obesity is an independent risk factor for chronic heart failure in older people. J Am Geriatr Soc 54: 413–420. 16551307 10.1111/j.1532-5415.2005.00624.x

[36] Nicklas BJ, Penninx BW, Cesari M, Kritchevsky SB, Newman AB, et al (2004) Association of visceral adipose tissue with incident myocardial infarction in older men and women: the Health, Aging and Body Composition Study. Am J Epidemiol 160: 741–749. 15466496 10.1093/aje/kwh281

[37] Wang J, Gallagher D, Thornton JC, Yu W, Horlick M, et al (2006) Validation of a 3-dimensional photonic scanner for the measurement of body volumes, dimensions, and percentage body fat. Am J Clin Nutr 83: 809–816. 16600932 10.1093/ajcn/83.4.809 PMC2723741

[38] Moyer VA (2012) Screening for and Management of Obesity in Adults: U.S. Preventive Services Task Force Recommendation Statement. Ann Intern Med. 10.7326/0003-4819-157-5-201209040-00475 22733087

[39] Wilson JP, Mulligan K, Fan B, Sherman JL, Murphy EJ, et al (2012) Dual-energy X-ray absorptiometry-based body volume measurement for 4-compartment body composition. American Journal of Clinical Nutrition 95: 25–31. 22134952 10.3945/ajcn.111.019273 PMC3238462

[40] Petrescu L, Strungaru CA, Mihailescu DF, Salistean A, Niculescu C, et al (2012) 3D Body Scanning Technology, A Method For Assessing Early Risk Of Diabetes. Proc Rom Acad 1: 3–8.

[41] Micklesfield LK, Goedecke JH, Punyanitya M, Wilson KE, Kelly TL (2012) Dual-energy X-ray performs as well as clinical computed tomography for the measurement of visceral fat. Obesity (Silver Spring) 20: 1109–1114. 22240726 10.1038/oby.2011.367 PMC3343346

[42] Kaul S, Rothney MP, Peters DM, Wacker WK, Davis CE, et al (2012) Dual-energy X-ray absorptiometry for quantification of visceral fat. Obesity (Silver Spring) 20: 1313–1318. 22282048 10.1038/oby.2011.393 PMC3361068

